# Influence of alkali treatment in enhancing crystallinity and breaking force of pineapple leaf fiber

**DOI:** 10.1038/s41598-025-19991-8

**Published:** 2025-10-15

**Authors:** Dharanendra Y T, Jamaluddin Hindi, Gurumurthy B M, Muralishwara K

**Affiliations:** Department of Mechanical and Industrial Engineering, Manipal Institute of Technology, Manipal Academy of Higher Education, Manipal, Karnataka 576104 India

**Keywords:** Pineapple leaf fiber (PALF), Alkali treatment, Sodium hydroxide, XRD, FTIR, SEM, Mechanical properties, Breaking force, Chemistry, Engineering, Materials science

## Abstract

The study investigates the effect of alkali treatment on pineapple leaf fiber (PALF) woven mats. The woven mat was chemically treated with varied concentrations (3, 6, and 9% w/v) of sodium hydroxide (NaOH) solutions for different exposure times (30, 60, and 90 min), making a total of 9 experiments. The structural, morphological, and chemical properties of untreated and alkali-treated specimens were investigated by using X-ray diffraction (XRD), Scanning Electron Microscopy (SEM), and Fourier Transform Infrared Spectroscopy (FTIR) respectively. The mechanical property was assessed through the breaking force analysis. The XRD result indicated that the fiber mat treated with NaOH of 6% w/v concentration exposed to 30 min yields a crystallinity index (CI) of 63.95% and a crystallite size (CS) of 7.05 nm. The FTIR analysis helped to identify chemically active groups involved in PALF and the characteristic absorption peaks associated with partial and complete removal of wax and other impurities. SEM results quantitatively indicated the elimination of amorphous components from the surfaces. The specimen with the highest CI of 63.95% exhibited the maximum breaking force of 356.92 N. The results indicate that the increase in CI and CS not only improves the mechanical properties but also helps in stronger interfacial bonding in polymer composite applications.

## Introduction

Fiber-reinforced polymer composites (FRPCs) have emerged as one of the most significant classes of materials in current engineering applications because of their excellent combination of low weight, corrosion resistance, and mechanical strength^1^. These materials are fabricated by polymer matrix, commonly thermosetting or thermoplastic, with reinforced fibers, which give strength and stiffness to the composite materials. Fibers make up most of a composite’s volume and are a significant form of reinforcement^2^. In composites, the matrix shares most of the loads on the fibers. FRPCs are extensively used in aerospace, automotive, marine, sports equipment, and construction sectors^3^.

Synthetic fibers, such as glass fibers, aramids, and carbon fibers, are generally used for reinforcement due to their advantages, namely high mechanical properties, good thermal stability, and superior stiffness^4^. Using these fiber reinforcements led to the fabrication of high-performance composites with superior strength-to-weight ratios. However, even though the synthetic fibers have many superior advantages, they also have drawbacks. Mainly, the production of these fibers depends on non-renewable petroleum resources, and they are non-biodegradable and leading to environmental concerns regarding waste management and pollution. Additionally, some fibers are associated with high costs^5^.

These drawbacks of the synthetic fibers prompted researchers to explore environmentally sustainable and inexpensive alternative materials. In this contest, natural fibers emerged as a promising reinforcement for polymer composites. Natural fibers are an appealing ecological substitute for synthetic fibers used in composites because they are less expensive, renewable, low-density, and have moderate mechanical properties^6–9^.

The natural fibers are derived naturally from plants, animals, and minerals. Natural fibers, apart from being used in composites in recent times, have been used since ancient times, such as construction materials, ropes, cloths, textiles, paper, etc. Among these three natural fibers, the plant-based natural fibers gained maximum interest for composite applications because of their wide availability, lower weight, and lower cost^2,10,11^.

Plant-based natural fibers are derived from various parts of the plant, including the stem, husk, seeds, and leaves. Sisal, flax, coir, bamboo, jute, and Pineapple Leaf Fiber (PALF) are some of examples of plant-based natural fibers^10,12^. Among them, PALF has high cellulose content, good mechanical strength, and modulus, making it a promising candidate for load-bearing applications^6,7,11^. The disadvantages of PALF are higher water absorption because they contain abundant hydroxyl (-OH) groups in their cellulose structure, which make hydrogen bonds with water molecules, and this hydrophilic nature makes poor fiber-matrix interfacial binding^13^. Various chemical treatments have been used to modify the surface of natural fibers and enhance their adhesion with the polymer matrix. The chemical treatment reduces the water absorption by removing non-cellulose content such as hemicellulose, lignin, and other surface impurities^14^. These treatments also roughen the surface of the fibers and increase the interfacial bonding with matrix, which improves the overall strength, durability, and stiffness. The various chemical treatments include alkali, silane, plasma, acetylation, permanganate, silane coupling agents, and peroxide treatments. Among the chemical treatments listed above, alkali (NaOH) treatment is the most common and simplest method^15^.

NaOH treatment involves soaking natural fibers in a NaOH solution at a specific concentration and temperature for a specific period. The mechanism of action of NaOH can be well explained with the help of the chemical Eq. (1)^16^

1 $${\text{Fiber}}{-}{\text{OH }} + {\text{ NaOH }} \to {\text{ Fiber}}{-}{\text{O}}{-}{\text{Na }} + {\text{ H}}_{{\text{2}}} {\text{O}}$$

Several studies have investigated the effect of alkali treatment on PALF. The focus is on how NaOH treatment enhances the mechanical and structural properties of PALF by improving fiber-matrix adhesion and removing non-cellulosic components.

M Asim et al. have investigated the physical and mechanical properties of alkali-treated PALF. The fibers were treated chemically with 3% and 6% NaOH for durations of 3, 6, 9, and 12 h. The 6% treated fiber exhibited maximum tensile properties compared to the untreated one, and SEM images display a clear surface with a rough texture. Soaking times beyond 6 h hurt the fibers, resulting in a decline in properties^17^. Eric Worlawoe Gaba et al. investigated mechanical and structural characteristics of PALF. The fibers were treated with 1%, 3%, 6%, and 9% wt% of NaOH for 60 min. The 6% treated fiber exhibited a crystallinity index (CI) of 74% and a crystallinity size (CS) of 24 nm, which directly affects the mechanical properties of PALF. The tensile strength of the 6% treated PALF exhibited 1620 MPa when compared with the untreated fiber of 63 MPa^18^. Bhoopathi et al. investigated the alkali-treated hemp fiber with epoxy matrix composites shows a significant tensile strength, flexural and impact strength of 48.3 MPa, 0.52 KN, and 8.62 KL/mm^2^, respectively, compared with untreated hemp^19^. Mysamy K have researched on the agave fiber treated with alkali and embedded in an epoxy matrix exhibited significant enhancements in tensile strength (270 MPa), flexural strength (3.85 GPa) and impact strength (1.53 J), as reported in reference^20^. Hu and Lim investigated the alkali-treated natural fiber hemp was used to prepare a composite with an epoxy matrix, showing improved mechanical properties compared to the untreated fiber. The composites treated with 40% alkali exhibited a tensile strength of 54.4 MPa and a flexural strength of 112.7 MPa, respectively^21^. Seki Y researched on the jute was chemically treated by the alkali method with 5% NaOH for 2 h and composites with epoxy matrix. The tensile properties were increased by 8% compared to untreated jute, and noted 69 MPa. Similarly the flexural strength and its modulus were increased by 10–15.9% respectively^22^. Lopattananon et al. investigated the PALF that underwent various chemical treatments such as alkali treatment, AN grafted, Acetylated PALF, and bleached fiber. Among all the AN grafted PALF with PP polymer matrix exhibits a good mechanical property. The tensile strength and flexural strength were 33.3 MPa and 49.9 MPa, respectively, compared to all other treated PALF^23^. Simamora et al. investigated the natural fibers, including snake plants, ferns, rice straw, coconut husk, and palm fiber were used as reinforcement in a polypropylene matrix. All these fibers were used to create composites after undergoing a 5% alkali treatment. The research work noted that the alkali-treated fibers enhanced the mechanical properties, especially the rice straw fiber with the highest tensile strength and flexural strength of 7.9 MPa and 139.6 MPa, respectively^24^. The review on chemical treatment, as summarised here, shows the structural and morphological changes in PALF, which confirms improving their crystallinity properties, which directly impacts the breaking force in composite materials^25–27^.

The research also reveals a significant gap in understanding the effect of alkali treatment on PALF woven mat. The previous studies provide the effect of NaOH treatment on single pineapple leaf fibers. The current research is on the PALF woven mat, with a proper investigation of how varying NaOH concentrations and treatment durations affect the mechanical and surface properties. This research aims to correlate crystallinity (CI), crystallite size (CS), and mechanical performance, contributing to optimized natural fiber composites.

## Materials and methods

### Materials

Pineapple leaf fiber was obtained from Gogreen Products, Chennai. It is a bidirectional woven mat of 300 GSM, and Sodium hydroxide (NaOH) pellets were purchased from Raman Lab Equipment’s and Chemicals, Udupi, Karnataka, India.

### NaOH treatment of PALF

The procured PALF mats when treated with NaOH to remove non-cellulosic chemical components namely hemicellulose, lignin, wax, and other impurities and improve the bonding between the fibers and the matrix. Removal of non-cellulosic contents enhances re-organization and compacts the cellulose chain, thereby enhancing the natural fiber’s crystallinity. An increase in crystallinity also enhances adhesion with the epoxy matrix and breaking force of the fiber. Therefore, as crystallinity is an independent parameter, it is important to optimize it concerning the input process factors/parameters. Two key input parameters were identified: NaOH concentration and exposure time. These parameters influence the extent of fiber modification, interaction with chemicals, and the overall treatment process and fiber characteristics.

The treatment was carried out with room temperature of 28 ± 2 °C and RH 60–75% with a freshly prepared NaOH solution in distilled water. Once the treatment was finished the treated fibers are thoroughly washed in running tap water followed by distilled water until neutral pH value is reached then dried in sunlight for 24 h.

**Fig. 1:**
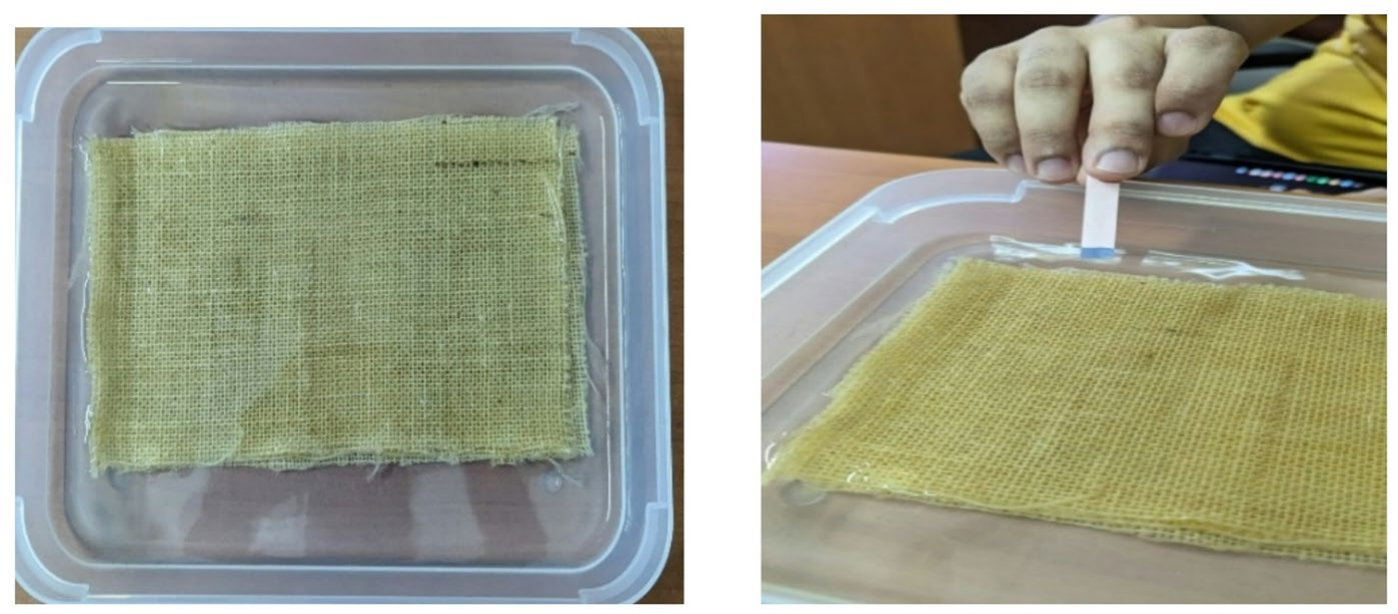
NaOH treatment of PALF mat.

The set of experiments on fibers with varying NaOH concentrations and times is presented in Table 1. Figure 1 illustrates the alkali treatment of procured PALF fibers.

**Table 1:** NaOH treatments on fiber.

Specimens number	Specimens name	NaOH % (w/v)	Time in min
1	S1-3 N-30 M	3	30
2	S2-6 N-60 M	6	60
3	S3-9 N-90 M	9	90
4	S4-3 N-60 M	3	60
5	S5-6 N-90 M	6	90
6	S6-9 N-30 M	9	30
7	S7-3 N-90 M	3	90
8	S8-6 N-30 M	6	30
9	S9-9 N-60 M	9	60
10	S10-UNT	Untreated	

The NaOH solution is neutralized by adding weak acids and reaches normal pH levels before disposal to promote environmental sustainability.

### Characterization of PALF

#### X-ray diffraction (XRD) of PALF

The XRD characterization of the PALF mat was performed using a Rigaku Miniflex 600 (5th Gen) diffractometer equipped with an X-ray source operating at up to 40 kV and 15 mA, and a nickel filter. The measurements were carried out at room temperature over a 2θ range of 10° to 80°, with a scanning speed of 2° per minute. Although the instrument is capable of bulk and thin-film analysis and supports a wider measuring range of 10°–130°, only the specified range was used for this study.

Crystallinity Index (CI) was calculated from the XRD plot from the XRD characterization according to Segal’s equation. The Segal equation is commonly employed to identify the CI of cellulose materials, which can impact the mechanical properties of fiber-embedded composites. The crystallinity index is calculated based on X-ray diffraction (XRD) data^28^

2 $${C}\text{I}{\%}=\frac{{\text{I}}_{002}-{\text{I}}_{\text{a}\text{m}}}{{\text{I}}_{002}}\text{*}100$$

where, CI is the crystallinity index.

I_002​_ is the intensity of the diffraction peak corresponding to the crystalline regions of cellulose or maximum diffraction peak intensity. (usually near 2θ = 22.6°).

I_am_​ is the intensity of the diffraction at a minimum between the amorphous peak and the next peak (usually near 2θ = 18°), representing the amorphous regions.

XRD characterization was also employed to calculate the crystallite size (CS) as per Scherrer’s equation shown below (3).

3 $$\:\text{C}\text{r}\text{y}\text{s}\text{t}\text{a}\text{l}\text{l}\text{i}\text{t}\text{e}\:\text{S}\text{i}\text{z}\text{e}\:\left(\text{C}\text{S}\right)=\frac{\text{K}{\uplambda\:}}{{\upbeta\:}\text{c}\text{o}\text{s}{\uptheta\:}}\:$$

where, K = 0.9 provides Scherrer’s constant in this case.

Here, λ stands for wavelength which is equal to 0.154 nm.

β is the peak’s entire breadth at half maximum also called Full Width Half Maximum (FWHM). FWHM is in radians and *θ* is half of the range of 2*θ* under the crystalline peak in radians^28^.

#### Scanning electron microscope (SEM) analysis

The SEM characterization of the PALF mat was conducted using an EVO MA18 with an Oxford EDS (X-act) machine, achieving a magnification from 1× to a maximum of 100,000×. Before SEM analysis, a thin layer of gold is applied to the specimens when characterizing non-conductive materials such as natural fibers or polymers. The gold sputtering of the PALF was done before SEM characterization with 10 mA for 60 s under 0.05 mbar. This process enhances imaging by reducing charge effects in the fibers or matrix, minimizing noise and improving resolution.

#### Fourier transform infrared spectroscopy (FTIR)

Fourier Transform Infrared (FTIR) spectra were performed by using a Perkin Elmer spectrometer with transmittance mode at a resolution of 2 cm^− 1^ throughout the range of 400–4000 cm^− 1^. FTIR characterization is employed to identify the chemical groups and bonding in alkali-treated and untreated PALF.

#### Breaking force testing of PALF mat

**Fig. 2:**
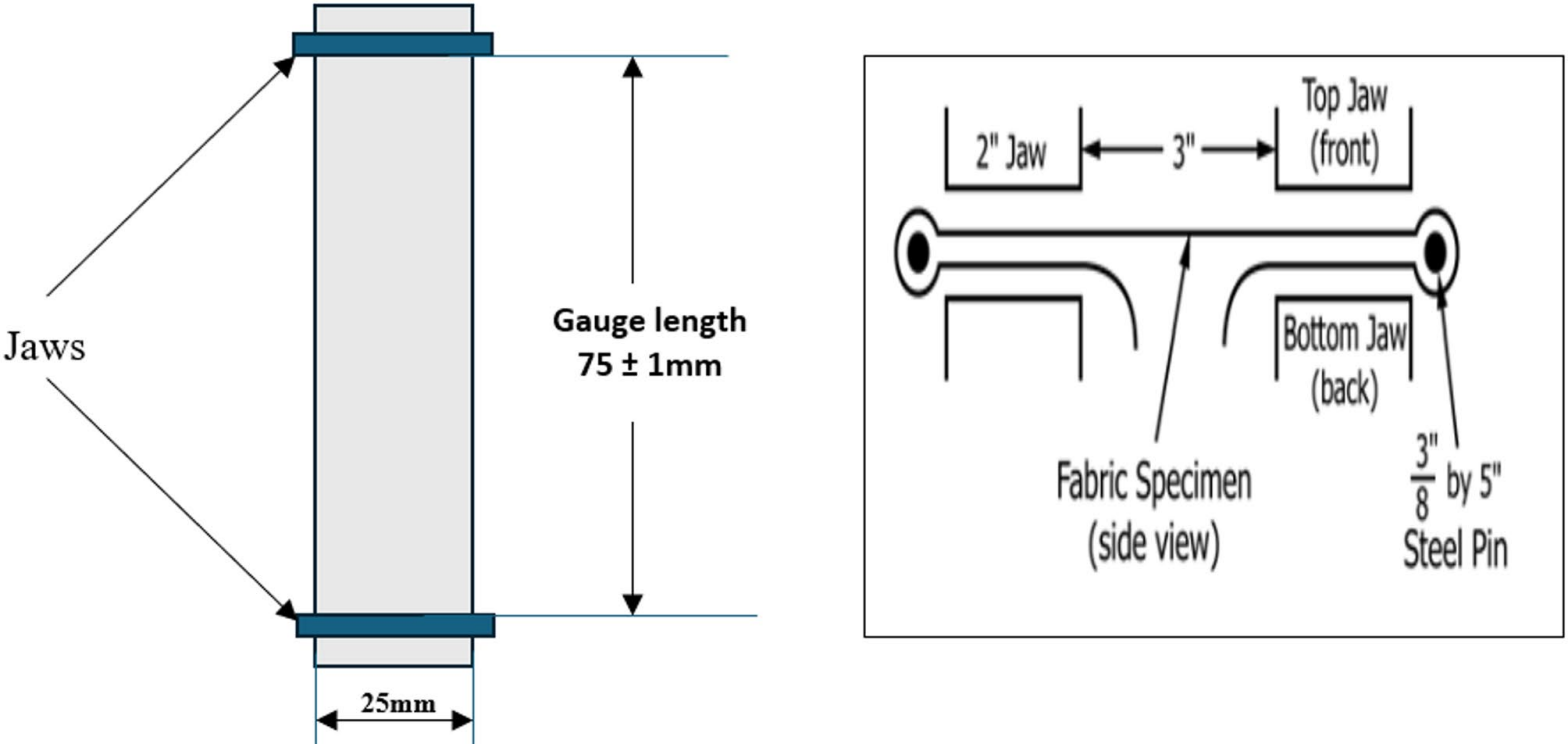
(**a**) Illustration of the specimen sketch and placement of the PALF mat as per ASTM D5035-11. (**b**) Side view of the fabric orientation^30^.

**Fig. 3:**
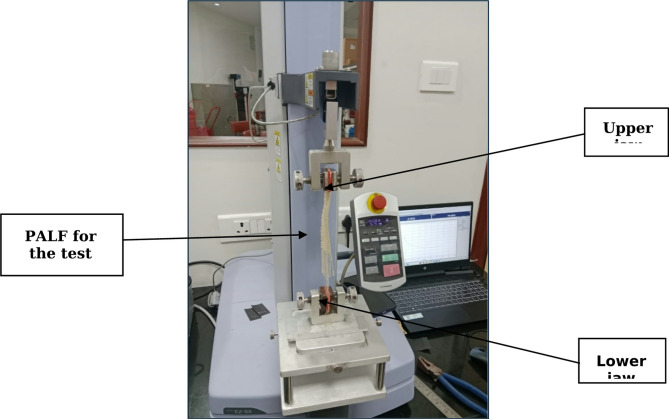
A digital tensile tester was used for the testing process.

The breaking force of NaOH-treated and untreated PALF was performed as per ASTM D5035-11 (strip method) and Fig. 2a,b shows the illustration of the specimen sketch and placement of the PALF mat as per ASTM D5035. The breaking force is tested using a Shimazdu EZ-SX digital tensile tester, as shown in Fig. 3. The CI of cellulose materials can affect the mechanical properties of fibers. Each specimen underwent three trials while conducting the breaking force test. The PALF mats were cut into 150 mm length and 25 mm width as per the ASTM standards^29^.

## Results and discussion

### Effect of NaOH treatment on the crystallinity of PALF (XRD analysis: crystallinity and crystallite size)

The NaOH treatment of PALF enhances crystallinity by removing all non-cellulose contents which leads to better alignment of cellulose chains, which strengthens the fiber^25^. Natural fibers contain both amorphous and crystalline structures and NaOH treatment dissolves the amorphous content resulting in an improvement in the CI in the fibers^31,32^. The removal of non-cellulose contents and impurities in the fibers makes the cellulose chain align uniformly and improves the surface roughness resettling in strong interlocking with the matrix^33^. The enhanced CI improves the mechanical properties due to its well-ordered cellulose and strong intra-molecular hydrogen bonding^34,35^. The XRD analysis was conducted for the PALF to calculate the CI and CS. The untreated PALF was subjected to various NaOH treatments, and each specimen underwent five trials to ensure the accuracy of the experiment.

**Fig. 4:**
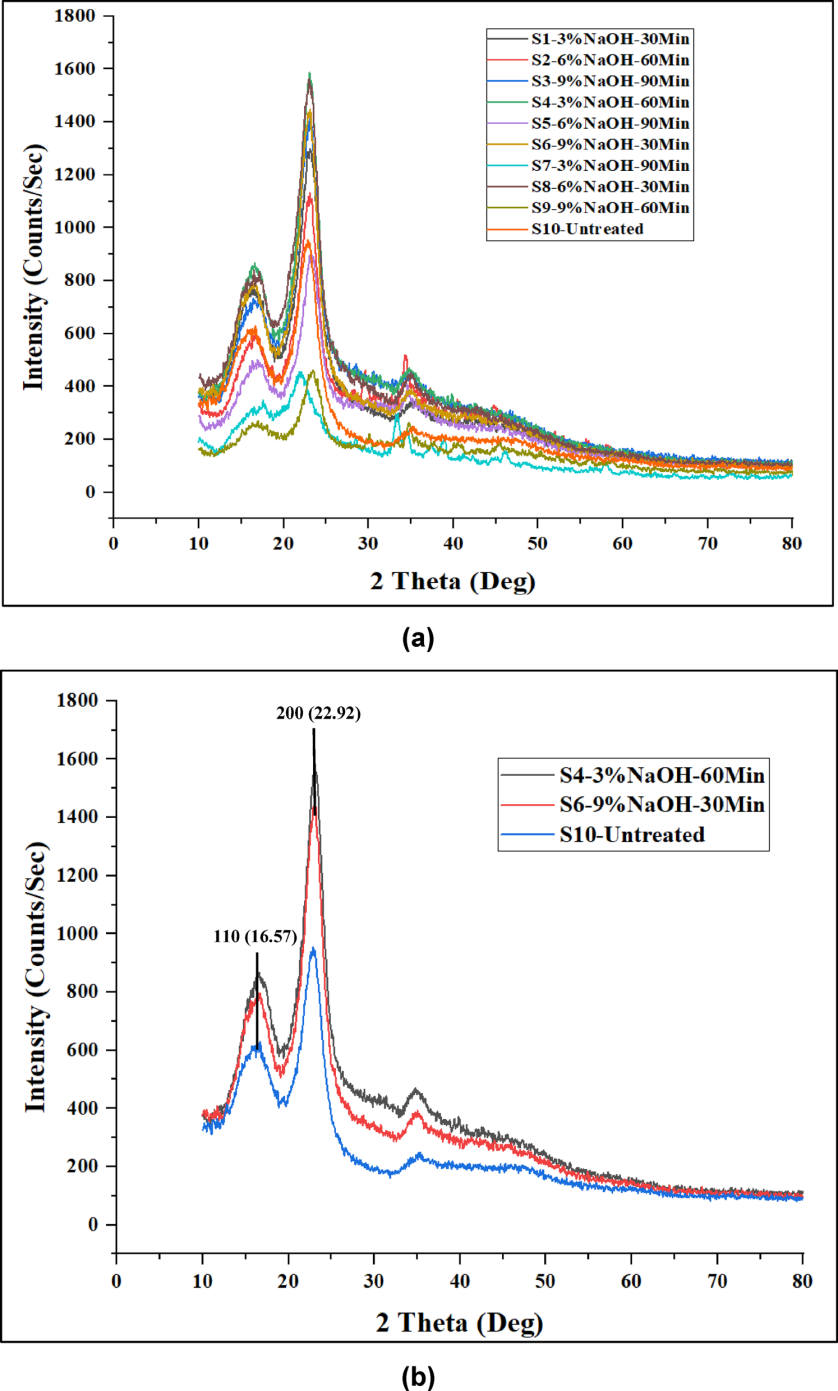
X-ray diffraction patterns of (**a**) all the specimenes, (**b**) S4, which has the highest crystallinity index, S6 lowest crystallinity index, and S10 (Untreated).

Figure 4 below shows X-ray diffraction patterns of (a) all the specimenes, (b) S4, which has the highest crystallinity index, S6 lowest crystallinity index, and S10 (Untreated).

The crystallinity index (CI) values of PALF treated with various concentrations of NaOH (3% w/v, 6% w/v, and 9% w/v) for different durations (30 min, 60 min, and 90 min) are compared with untreated PALF. At 30 min of treatment, the CI values are 60.45 for 3% w/v, 62.40 for 6% w/v, and 43.93 for 9% w/v NaOH-treated PALF. For 60 min, the respective CI values are 63.95 (3% w/v), 60.00 (6% w/v), and 54.18 (9% w/v). Similarly, at 90 min of treatment, the CI values are 62.63 for 3% w/v, 60.62 for 6% w/v, and 53.88 for 9% w/v, in comparison to 53.96 for untreated PALF. Based on these results, it can be observed that PALF treated with 6% w/v NaOH for 30 min, 3% w/v for 60 min, and 9% w/v for 90 min exhibit notable improvements in crystallinity when compared to the untreated specimen, indicating effective chemical modification under these conditions.

The results indicated that specimen S4, treated with 3% NaOH for 60 min, achieved the highest CI of 63.95%. This demonstrates significant improvements in the crystalline properties due to optimized treatment parameters. In contrast, specimen S6, treated with 9% NaOH for 30 min, exhibited the lowest CI of 43.93%, suggesting that increasing the NaOH concentration negatively affects the crystallinity of the PALF. The NaOH treatment with 9% w/v causes overly aggressive removal of lignin and hemicellulose and also degrades cellulose fibrils, leading to structural damage and reduced mechanical properties^36,37^.

**Fig. 5:**
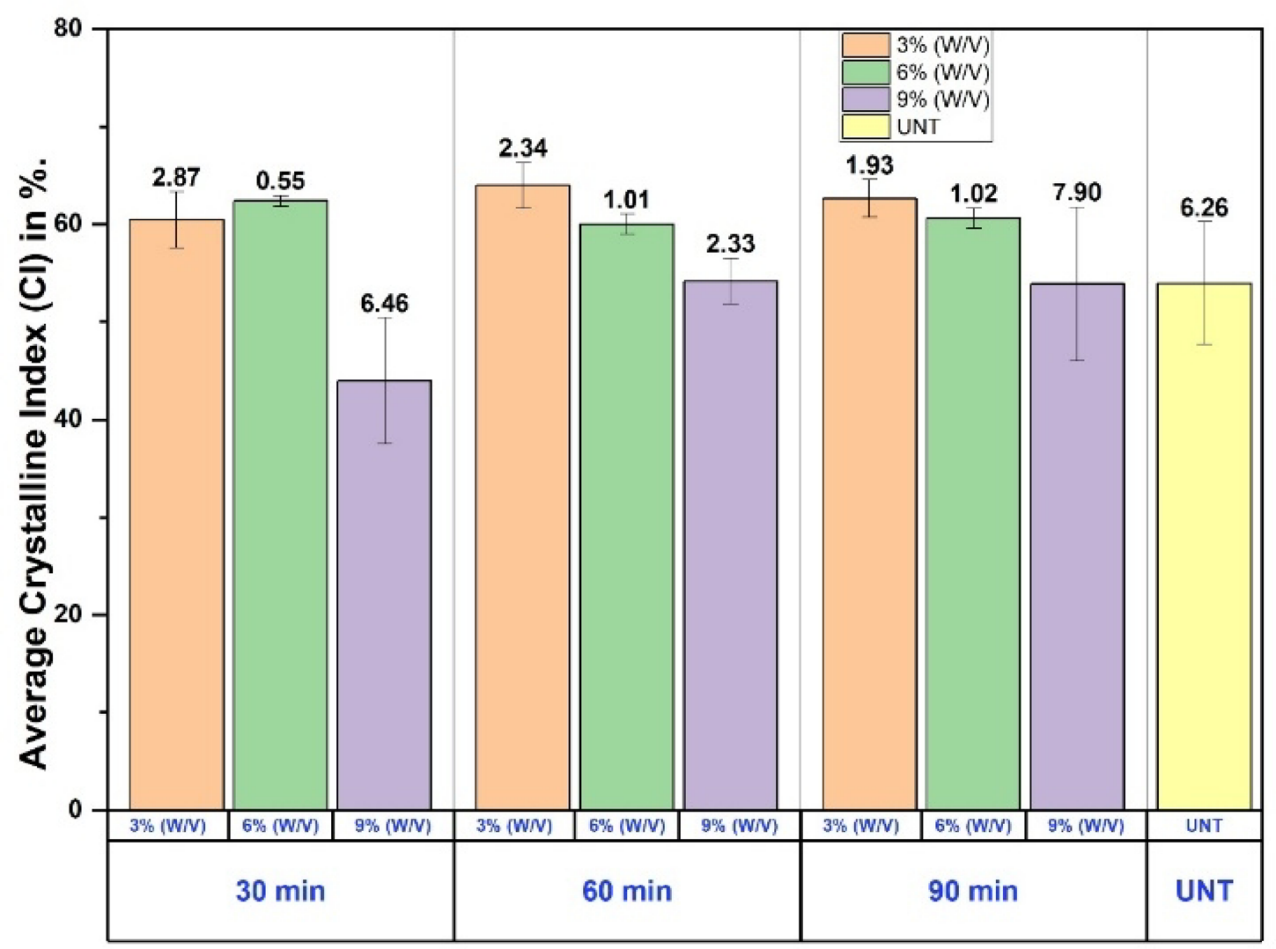
The average CI% and its standard deviation.

Overall, the CI of the highest-performing treated specimen S4 was considerably higher than that of untreated PALF, highlighting the effectiveness of chemical treatment in enhancing the structural properties of the fibers. Figure 5 below shows the average value of CI, and its standard deviation is plotted in a grouped bar chart. The bar chart is used to understand the deviation within the specimens and between the specimens of different concentrations of NaOH and duration.

**Fig. 6:**
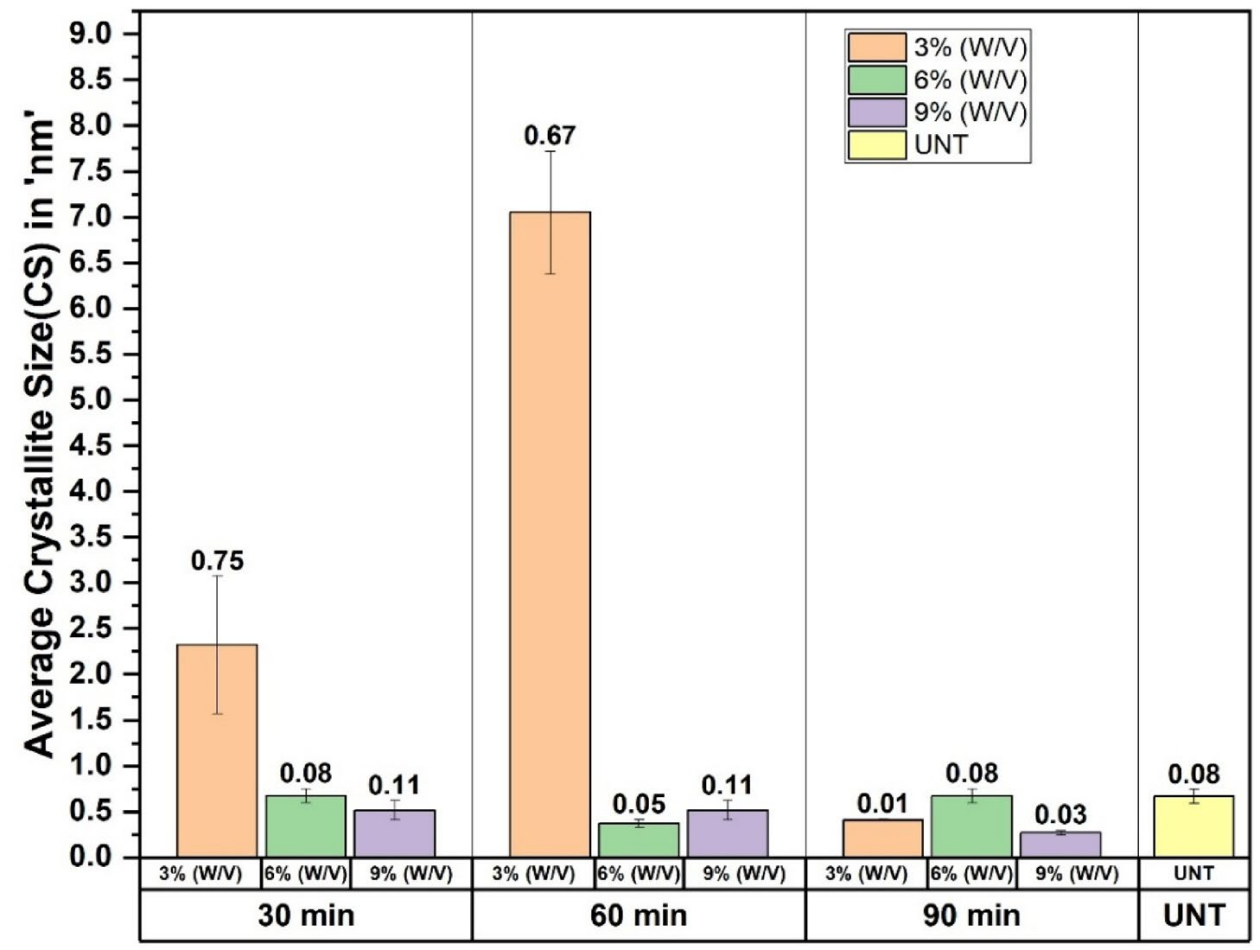
The average CS and its standard deviation.

The specimen S4, treated with 3% NaOH for 60 min, exhibited the highest CS of 7.05 nm when compared with the untreated one. Figure 6 below shows the average value of CS, and its standard deviation is plotted in a grouped bar chart. The bar chart is used to understand the deviation within the specimens and between the specimens of different concentrations of NaOH and duration.

### Impact of NaOH treatment on surface morphology of PALF

**Fig. 7:**
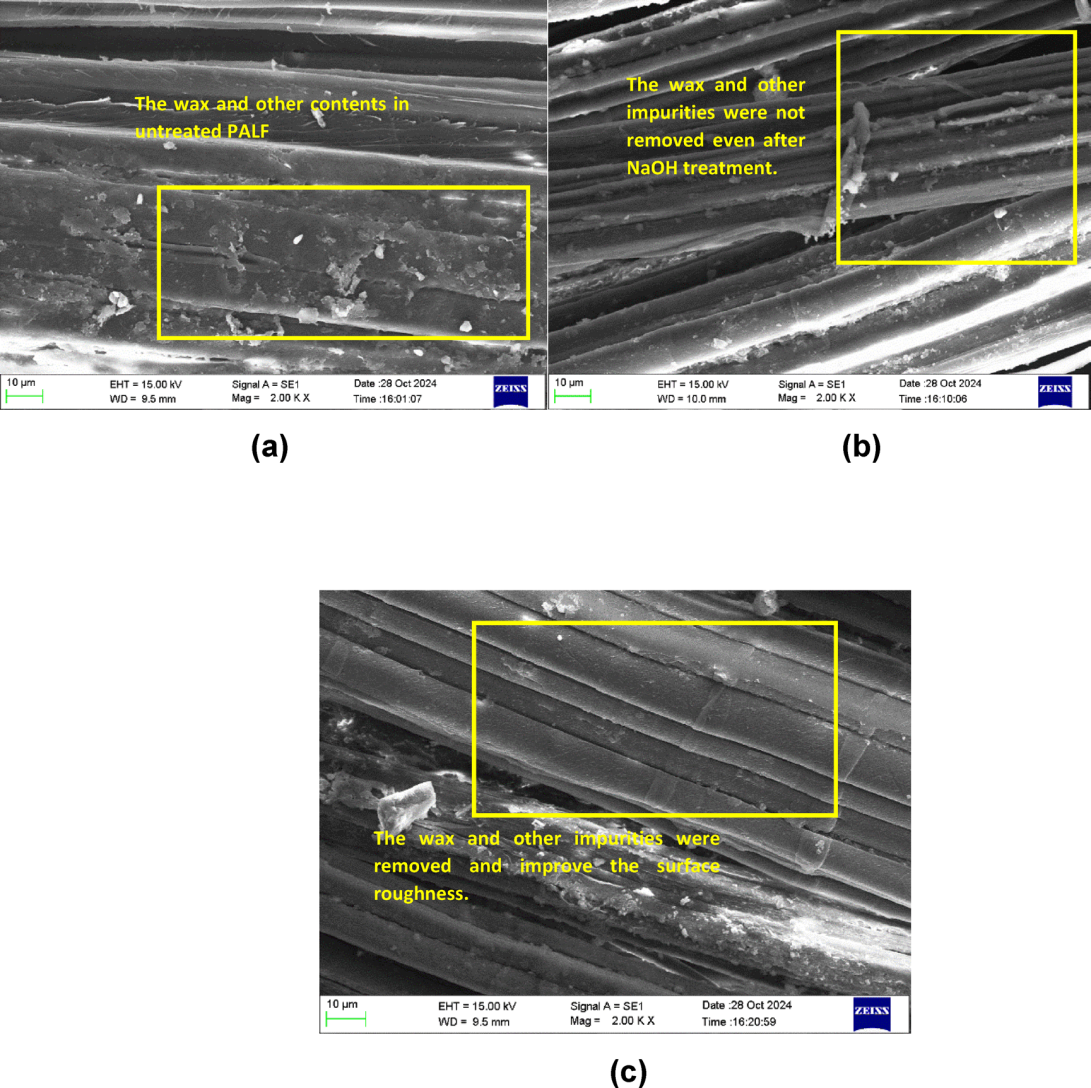
SEM image of (**a**) Untreated PALF, (**b**) Low crystallinity specimen S6-9 N-30 M, (**c**) High crystallinity specimen S4-3 N-60 M.

Once the crystallinity of PALF is determined, the surface roughness and presence/absence of impurities in the fibers are evaluated by SEM. The SEM characterization was done for the three specimens: specimen 4(S4), which has the highest crystallinity, specimen 6 (S6), which has less crystallinity, and untreated PALF. Figure 7a–c shows the SEM images of S4, S6, and untreated PALF(S10).

The above SEM images show the elimination of surface impurities and the effect of NaOH treatment on PALF. The hemicellulose, lignin, wax, and other impurities were removed during the NaOH treatment, which also altered the rougher topology of the fibers. This rougher surface increases the bonding with the matrix. The enhanced roughness of the fibers directly affects the mechanical interlocking with the matrix, which results in better stress transfer during tensile loading^31,33,34,38,39^. Figure 7c shows the clear surface roughness and neat removal of impurities compared to the untreated PALF (S10) and specimen S6, which is shown in Fig. 7a,b.

### FTIR analysis

**Fig. 8:**
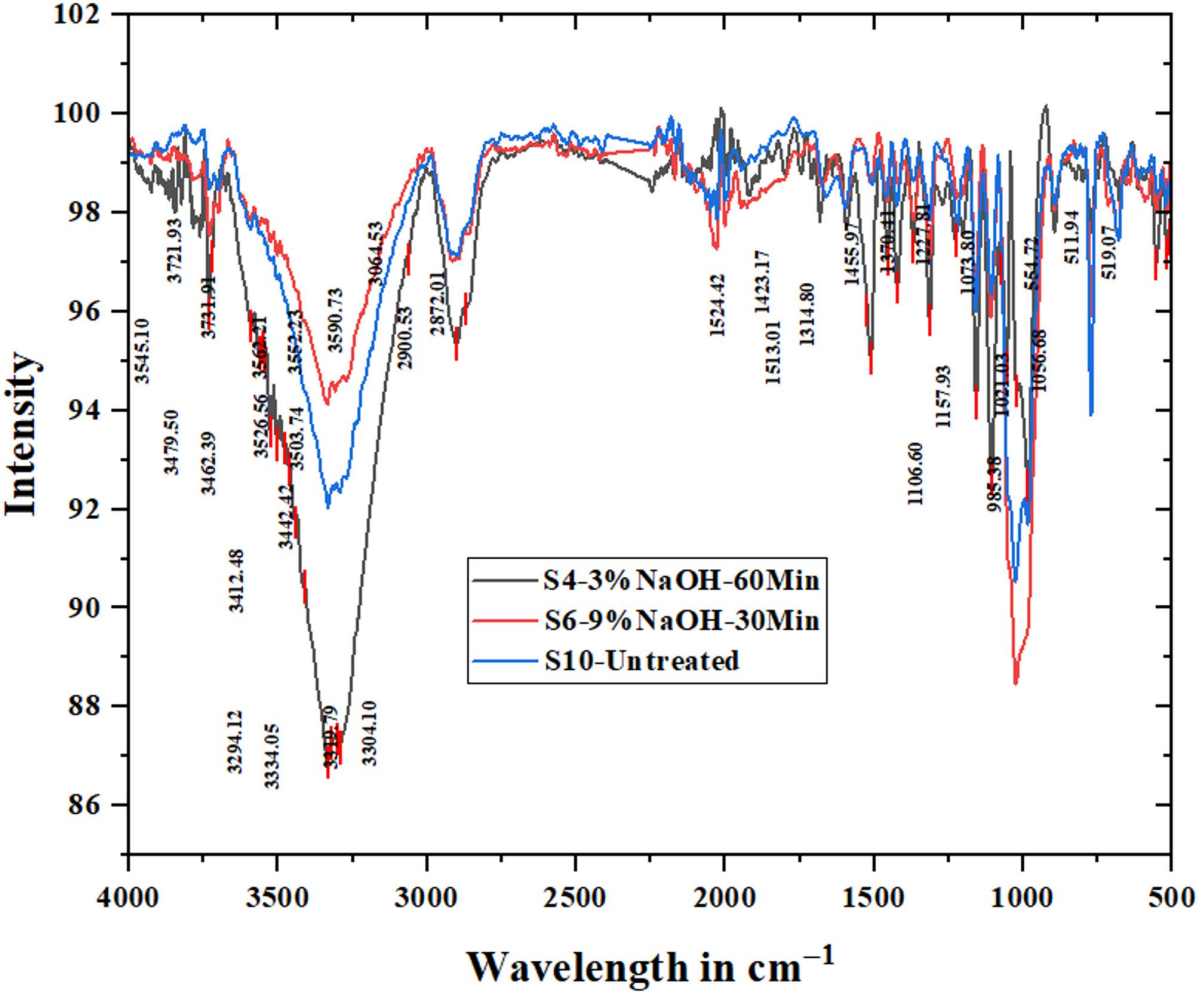
FTIR analysis of the combined graphs of S4, S6, and S10 along with their peaks.

Table 2 shows the list of the specimens which are high in CI (S4-3 N-60 M), low CI (S6-9 N-30 M), and untreated PALF(S10-UNT) and their peaks of O–H stretching (3334–3294 cm^−1^), C–O stretching (1026–1106 cm^− 1^) and lignin (985.38–772.91 cm^− 1^). FTIR characterization reveals that the specimen S4 with a CI of 63.95 has maximum cellulose exposure with fewer impurities at the O-H stretching peak at 3334.05 cm^− 1^, C–O stretching peak at 1106.6 cm^− 1^, and lignin peak at 985.38 cm^− 1^ when compared with other two specimen S6 with 43.92 CI and untreated PALF^35,40–43^. This characterization gives the effectiveness of NaOH treatment of PALF in changing the fiber structure with significant implications for the mechanical properties and adhesive bonding between the fiber and polymer matrix in composite materials^44–46^. Figure 8 shows the FTIR analysis of the combined graphs of S4, S6, and S10 along with their peaks.

**Table 2:** FTIR analysis and its observations.

Performed specimens	1st Peak in cm^− 1^ (O-H Stretching)	2nd Peak in cm^− 1^ (C-O Stretching)	3rd Peak in cm^− 1^ (Lignin/Impurities)	Observations
S10-UNT (Untreated-Baseline)	3332.62	1026.24	772.91	Hemicellulose and lignin present, lower crystallinity
S6-9 N-30 M (NaOH Treated-Low CI)	3334.05	1025.31	772.91	Better crystallinity, maximum cellulose exposure
S4-3 N-60 M (NaOH Treated-High CI)	3334.05	1106.6	985.38	Better crystallinity, maximum cellulose exposure

### Breaking force testing of PALF mat

**Fig. 9:**
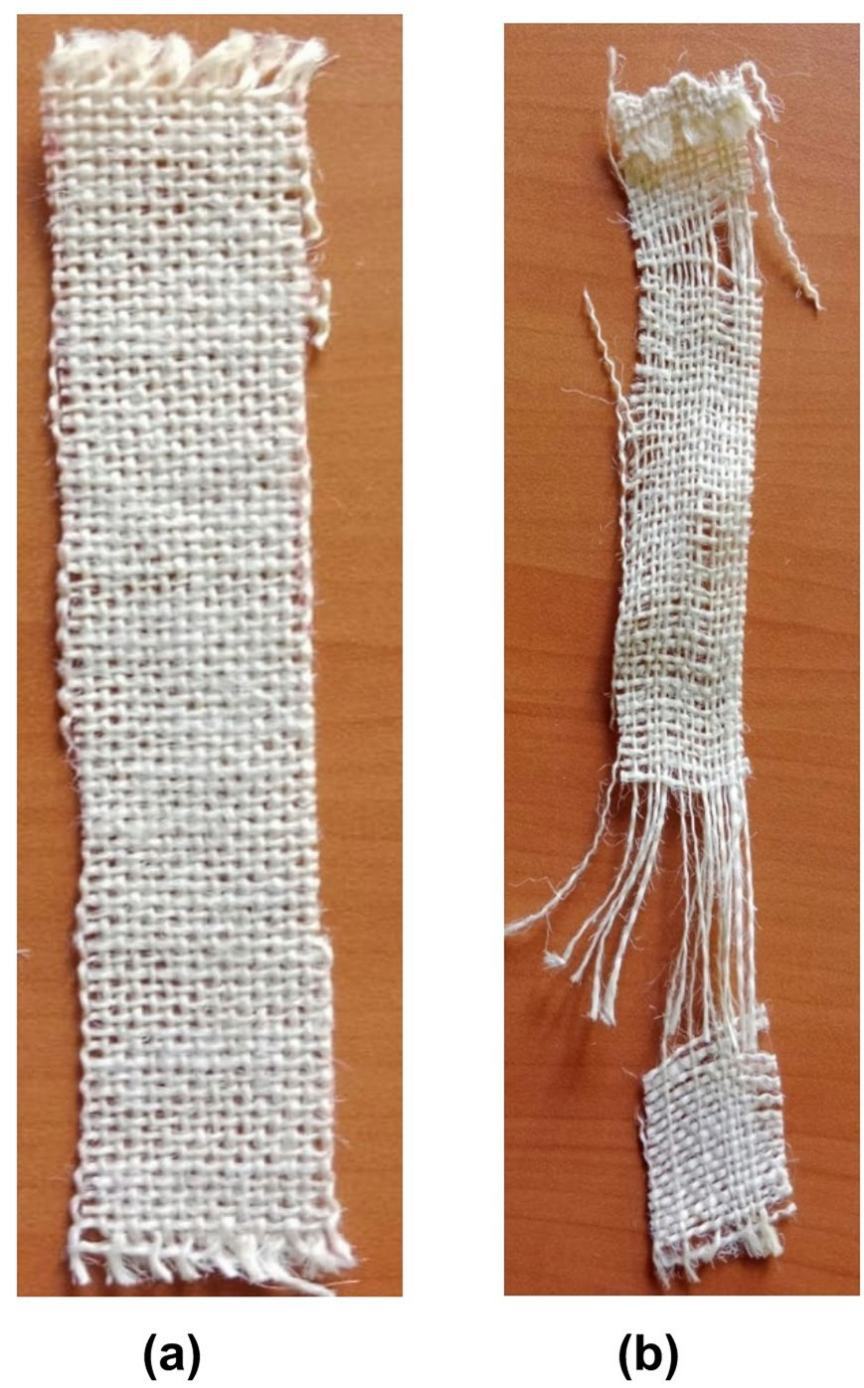
Images of PALF mat (**a**) Before the breaking force test. (**b**) After the breaking force test as per ASTM D5035.

The breaking force testing was performed to evaluate the effect of crystallinity. The nine NaOH-treated (S1, S2, S3, S4, S5, S6, S7, S8, and S9) and one untreated (S10) PALF specimens were subjected to a breaking force test for three trials. The results showed a direct relationship between the CI and the breaking force properties of the chemically treated PALF. Table 3 below shows the CI and breaking force properties of the specimens. Figure 9a,b shows the PALF mat images before and after the breaking force test as per ASTM standards D5035.

**Table 3:** Average CI and breaking force properties of the PALF specimens with standard deviation.

Serial number	Specimens name	Average of CI in %	Average of breaking force in *N*	Standard deviation
1	S1-3 N-30 M	60.45	315.55	3.72
2	S2-6 N-60 M	60.00	278.58	4.71
3	S3-9 N-90 M	53.88	260.10	1.61
4	S4-3 N-60 M	63.95	356.92	6.18
5	S5-6 N-90 M	60.62	332.25	8.83
6	S6-9 N-30 M	43.93	248.25	5.23
7	S7-3 N-90 M	62.63	281.24	6.49
8	S8-6 N-30 M	62.40	300.54	12.20
9	S9-9 N-60 M	54.18	250.23	4.10
10	S10-UNT	53.96	260.22	4.10

The specimen S4 with the highest crystallinity shows the maximum breaking force of 356.92 N when compared to other treated specimens and untreated ones. Specimen S5, with a CI% of 60.62, gives the breaking force of 332.25 N, which is nearer to the S4. With low CI%, and untreated specimens S6 and S10 noted breaking force of 248.25 N and 360.22 N, respectively.

**Fig. 10:**
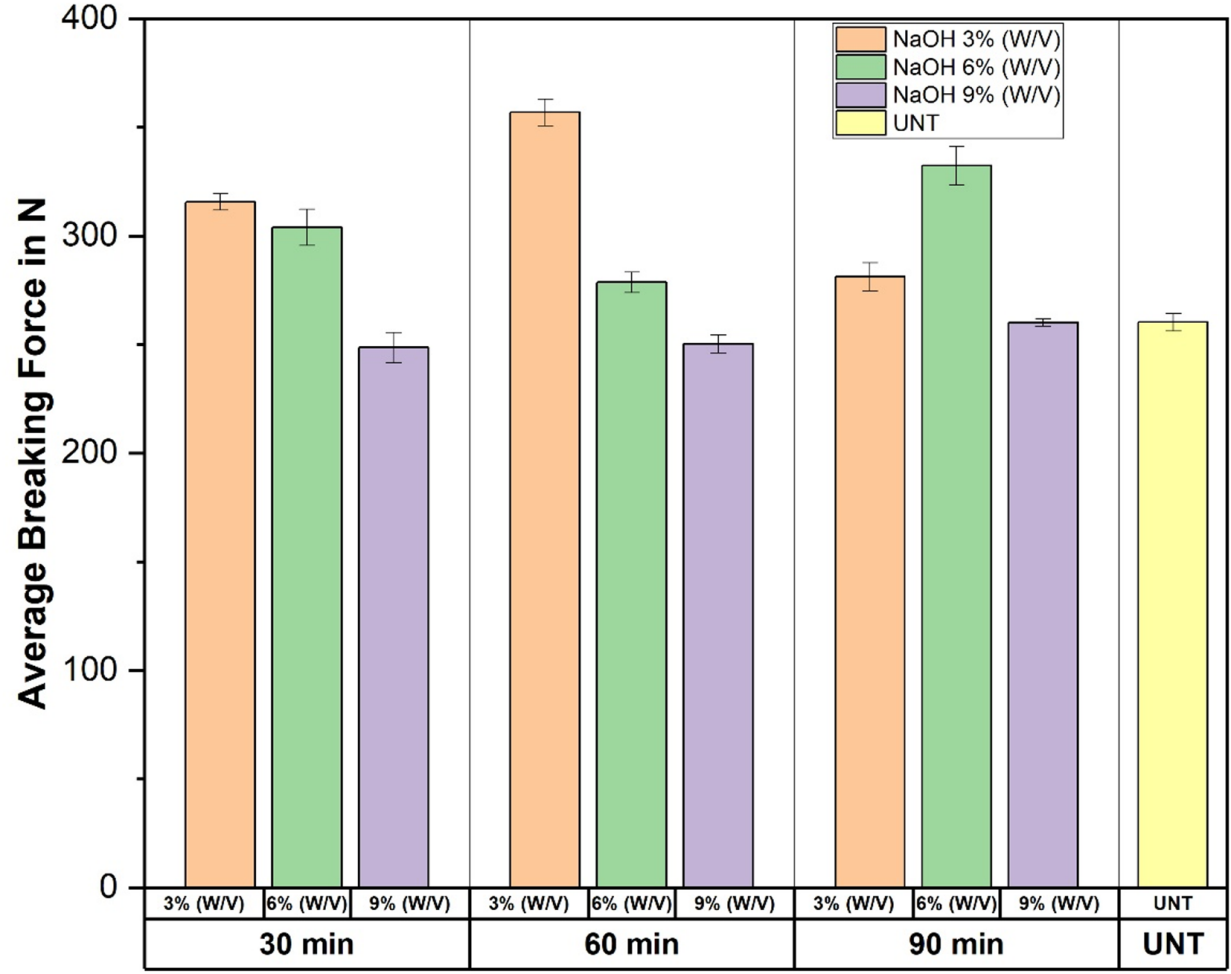
The average breaking force in N and its standard deviation.

Overall, it is concluded that the crystallinity index directly affects the mechanical properties of the PALF, and an enhancement in crystallinity will result in improved mechanical properties. Figure 10 shows the average breaking force with their standard deviations. The values obtained show some variation across the different specimens. This variation is expected due to the natural heterogeneity of PALF fibers and the manual preparation of woven mats. A lower (1.61 N) standard deviation reflects more uniform behaviour among the specimens, while a higher (12.20 N) standard deviation highlights greater variability in fiber properties and testing outcomes.

The increase in breaking force of PALF mats after NaOH treatment is mainly due to changes in the fiber structure. The treatment removes hemicellulose and lignin, which makes the fiber surface rougher and improves bonding with the matrix. It also raises crystallinity, which enhances stiffness and strength. At very high NaOH levels, however, the cellulose may get damaged, leading to reduced performance.

## Conclusion

In this present research work, the PALF woven mats were alkali-treated with 3, 6, and 9% w/v for 30, 60, and 90 min time durations. Different characterizations, such as XRD, SEM, and FTIR analysis, were performed to confirm the increase in crystalline properties and the removal of non-cellulose contents, including hemicellulose, lignin, and other impurities. The 3% NaOH with 60 min specimen (S4) exhibited the highest CI of 63.95 and CS of 7.05 nm when compared with the untreated, which was 53.96 and 0.66 nm. The increased crystalline properties of fibers exhibit the enhancement in the breaking force from 260.22 (Untreated specimen S10) to 356.92 N (S4). Also, an increase in NaOH treatment of 9% w/v with 30 min (S6) shows a negative impact on fibers, which exhibit the lowest CI of 43.93, CS of 0.51 nm, and breaking force of 248.25 N. In conclusion, the surface modification of the PALF woven mat provides comprehensive insights into optimizing the processing conditions necessary for effectively integrating this material with various polymer matrix systems.

## Data Availability

The datasets generated and/or analysed during the current study are not publicly available currently due to the reason that, it is part of Doctorial Studies of the first author, but are available from the corresponding author on reasonable request after the award of Ph.D.
